# Assessing the image of pharmacists and perceived public prospective on drive through pharmacy services in Saudi Arabia: A cross-sectional study

**DOI:** 10.1097/MD.0000000000048982

**Published:** 2026-05-29

**Authors:** Raeed Alanazi, Mohammed Almutairi, Mohammad K. Alharbi, Muteb Abdullah Saqer Alfutaymani, Marzouq Daije S. Alotaibi, Naji Alqahtani, Wajid Syed

**Affiliations:** a Department of Nursing Administration and Education, College of Nursing, King Saud University, Riyadh, Saudi Arabia; b Medical Surgical Nursing Department, College of Nursing, King Saud University, Riyadh, Saudi Arabia; c Health Informatics Specialist, College of Nursing, King Saud University, Riyadh, Saudi Arabia; d Medical Laboratory Technician, College of Nursing, King Saud University, Riyadh, Saudi Arabia; e Department of Clinical Pharmacy, College of Pharmacy, King Saud University, Riyadh, Saudi Arabia.

**Keywords:** Drive throughBenefits, Dispensing, Drive through, Perceptions, Pharmacy services

## Abstract

Evaluating consumer expectations and perceptions of established services is crucial to assessing the quality of drive through pharmacy services. Therefore, this study aimed to assess public perceptions of pharmacist image and the perceived advantages and disadvantages in response to the establishment of drive through pharmacy services. A cross-sectional quantitative study was conducted among individuals living in Riyadh, the capital of Saudi Arabia, in 2024. The study took place from March to July 2024. The questionnaire consisted of 5 sections and a total of 28 items. The mean age of the respondents was 58.4 years (standard deviation = 10.4; range: 20–83 years). Approximately 60% of respondents believed that pharmacists maintain an appropriate balance between patient care and the business aspects of their profession. Moreover, 55.2% agreed that prescriptions are likely to be processed more quickly at drive through pharmacies compared with traditional settings. Notably, 88.4% (n = 296) recognized drive through pharmacies as particularly beneficial for serving individuals who are ill, elderly, or physically disabled. The mean score for the public image of pharmacists was 10.9 ± 2.1 (median = 11), while the mean perceived benefit score for drive through pharmacies was 26.5 ± 4.06 (median = 28). In contrast, the mean score for perceived drawbacks was 24.1 ± 5.4 (median = 24). Overall, the current study provided a preliminary insight into people’s perceptions of the role of pharmacists, as well as the benefits and drawbacks of drive through pharmacy services, which had not yet been published. A majority of respondents felt that pharmacists strike a good balance and prioritize patient health over commercial matters.

## 1. Introduction

More recently, the dispensing practice in pharmacy settings has been advancing drastically, with drive through pharmacies being one of the global advancements.^[1–3]^ According to literature, a number of hospitals across Saudi Arabia have opened drive through services to serve patients.^[2]^ The drive through pharmacy services are aimed at providing fast and convenient drug delivery to patients and customers by reducing overcrowding and long waiting hours.^[4]^ The concept of drive through pharmacies was first established in the United States of America by Walmart pharmacies in the late 1990s, and later other countries adopted this procedure.^[5,6]^ These pharmacies are mostly found on hospital premises or in private community outlets. For example, in Saudi Arabia, King Faisal Specialist Hospital and Research Center in Jeddah owns a drive through service.^[7]^ In the northwest of Saudi Arabia, King Salman Armed Forces Hospital has unveiled a drive through machine to dispense medicines electronically.^[7]^

The role of the pharmacist has dramatically changed over the years from drug dispensing to patient counseling.^[8–11]^ The success of pharmaceutical care provided by the pharmacist mainly depends on the patient’s trust and relationship with the pharmacist.^[11,12]^ In this scenario, patients grant authority to pharmacists to manage their health and well-being, while pharmacists accept responsibility for the well-being of the patients.^[12,13]^ Furthermore, pharmacies are the frontline healthcare providers available to patients and the public without any prior appointment for the sale and distribution of medicines. Pharmacist loyalty is crucial in the medical supplies and pharmacy business.^[14]^ Therefore, understanding how the public perceives pharmacists with the introduction of drive through pharmacy services is essential.

Even as pharmacists strive aggressively to provide essential healthcare services,^[15,16]^ it is important to consider the public’s perception of pharmacists offering drive through pharmacy services.^[17,18]^ The public’s view of healthcare services in the pharmacy sector is a good indicator of how well a service has been implemented, as it can be measured by patient satisfaction.^[18,19]^ Despite the benefits of drive through pharmacy services, literature has shown a lack of time for patient counseling at the drive through window.^[4,19]^ It is crucial to compare consumers’ expectations and perceptions with established services to assess the quality of drive through pharmacy services and make necessary improvements to better serve patients and the public. While drive through services offer convenience, there are concerns about health effects and limited opportunities for counseling.^[2,4,19]^ Studies have shown that drive through services restrict interaction between pharmacists and patients,.^[19,20]^ Furthermore, there have gap in this area more particular in Saudi Arabia.^[4,19]^ Therefore, it is important to conduct this investigation. This study aims to evaluate public perceptions of pharmacists’ image and the perceived advantages and disadvantages of establishing drive through pharmacy services in Riyadh, Saudi Arabia.

## 2. Method

### 2.1. Study design, setting, and population

A cross-sectional quantitative study, using a convenience sampling technique, was conducted among individuals living in Riyadh the capital city of Saudi Arabia, over a period of 4 months in 2024. Prior to data collection the Research Ethics Committee at King Saud University for human research in Riyadh Saudi Arabia, approved the study questionnaires (King Saud University-HE-23–852). Furthermore, all research steps were performed in accordance with the Helsinki guidelines for human research. In addition, before proceeding with the study informed consent was obtained from all respondents involved in the study. Consenting adults who were Saudi nationals, aged above 18 years, living in the capital region, able to provide informed consent and willing to complete the questionnaires were included. Those who did not agree to the study requirements were excluded from the study.

### 2.2. Sample size estimation

The minimum required sample size was estimated using the Raosoft sample size calculator, assuming a Riyadh population of approximately 7,821,000, a 95% confidence level, and a 5% margin of error. The calculation yielded a minimum target of 385 participants. This estimation was used as a reference to ensure an adequate number of responses for descriptive analysis. However, as convenience sampling was employed, the calculated sample size does not imply probability-based representativeness of the Riyadh population. A total of 390 individuals were approached to account for potential incomplete responses.

### 2.3. Data collection and questionnaire

The questionnaire used in this study was adapted from earlier published studies.^[19,21]^ Items were selected based on their relevance to the current study objectives and cultural context. The questionnaire (28 items) consisted of 4 main sections. The first section collected respondents’ sociodemographic information, including age, gender, marital status, educational level, and employment status (5 items). The second section assessed public perceptions of the professional image of community pharmacists following the introduction of drive through pharmacy services (3 items). This section included statements evaluating whether drive through services influence perceptions of pharmacists’ focus on patient care versus business interests. The third section-explored respondents’ opinions regarding drive through pharmacy services compared with traditional in-store pharmacy services (7 items). Items addressed issues such as dispensing speed, availability of pharmacists for consultation, provision of written information, quality of counseling, and overall convenience, particularly during pandemic situations. Responses in these sections were recorded using a 5-point Likert scale ranging from “strongly disagree” to “strongly agree.” The fourth section examined perceived benefits (6 items) and drawbacks (7 items) of drive through pharmacy services. Benefit-related items focused on accessibility, timeliness of medication supply, suitability for elderly, disabled, or sick patients, infection control, and reduction of pressure on healthcare facilities. Drawback-related items assessed concerns related to communication quality, dispensing errors, reduced counseling opportunities, financial costs, and potential decline in service quality.

**Figure 1.**
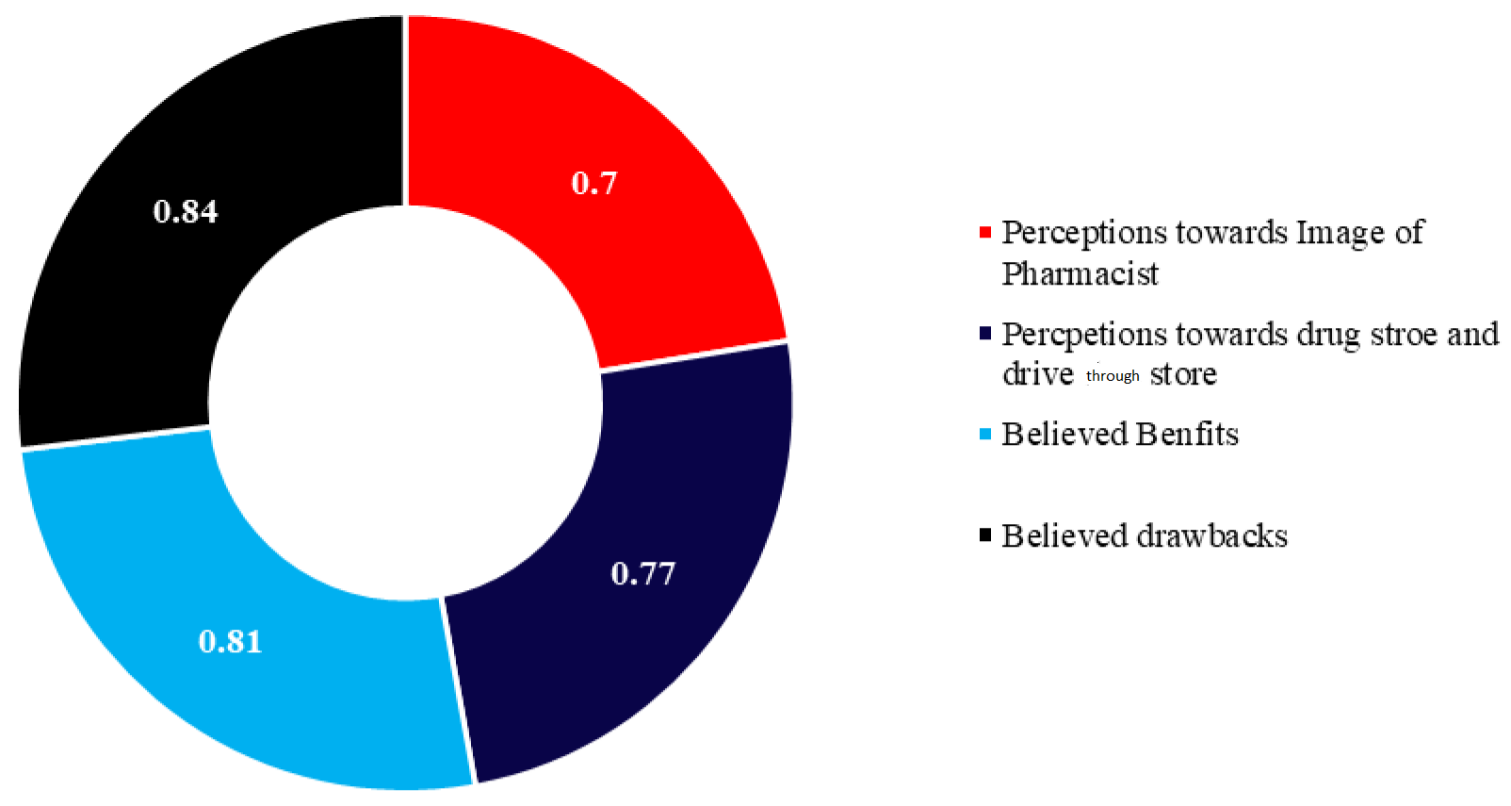
Internal consistency reliability of the study questionnaire assessed using Cronbach alpha

The initial draft of the questionnaire was translated from English into Arabic using a forward and backward translation procedure by bilingual experts proficient in both languages. The translated version of the questionnaires was later reviewed for clarity by 2 independent reviewers and pilot-tested among randomly selected respondents (n = 30). The pilot study aimed to identify any wording that could possibly hinder their understanding. The pilot findings were not included in the main findings. The internal consistency was assessed using Cronbach alpha, which was found to be 0.78, for all of the scale items (Fig. 1**).** These steps ensured that the instrument was both reliable and appropriate for our study population.

The final questionnaires were sent to the respondents using an electronic link generated by Google Forms (Google). The data collection was followed by a convenience sampling procedure. The targeted populations were approached using social media. Prior to filling out the questionnaires there was a statement about the significance of the study, confidentiality of the data, and residence status (living in Riyadh). Those who agreed to proceed were directed to the original questionnaires. The study took approximately 12 to 17 minutes to complete. Responses of participants were kept anonymous and confidential. To reduce the likelihood of duplicate responses, the online survey platform was configured to limit 1 response per device/IP address. In addition, responses were screened during data cleaning for identical entries and inconsistent patterns. Participants had the choice and the right to withdraw from filling out the questionnaire or ignore answering any question without justification.

### 2.4. Statistical analysis

The data were analyzed using the Statistical Package For Social Science 22.0 software program, descriptive statistics were used, calculating frequencies (n) and percentages (%) for quantitative variables and frequencies with percentages for categorical variables. The data, measured on an ordinal scale, were tested for normality using the Shapiro–Wilk test, which showed a significant deviation from normal distribution (*P* < .05). Therefore, nonparametric tests, including the Mann–Whitney *U* and Kruskal–Wallis tests, were applied to examine relationships between variables. All statistical tests were performed at a *P* value < .05 to be considered statistically significant.

## 3. Results

### 3.1. Characteristics of the study population

Of the 390 participants approached, 335 completed all items and were included in the final analysis (response rate 85.9%). Thirty-three responses were incomplete, and 22 did not meet inclusion criteria, therefore they were excluded from the study to ensure accuracy of the analysis. Among the respondents, the majority were male (232, 69.3%), with an age range of 58.4 years (standard deviation (SD) = 10.4, range 20–83). Thirty-four percent were between 21–25 years old. Most respondents (53.7%) were married, and the majority were educated, with only 13.1% declaring secondary education. Half of them were employed. Table 1 provides detailed demographic characteristics of the study population

**Table 1:** Characteristics of the study population (n = 335).

Characteristics	Frequency n (%)	Percentage (%)
Gender Male Female	232 103	69.3 30.7
Age (yrs) 21–25 26–30 31–35 36–40 41–45 > 46	114 64 79 47 22 9	34.0 19.1 23.6 14.0 6.6 2.7
Marital Status Single Married Divorced Widowed	138 180 9 8	41.2 53.7 2.7 2.4
Educational degree Secondary school University Doctorate	44 240 51	13.1 71.6 15.2
Employment Unemployed Employed Student House wives Retired	37 175 2 101 20	11.0 52.2 0.6 30.1 6.0

n = number of respondents, yrs = years.

### 3.2. Image of pharmacist with the drive though pharmacy

In this study, respondents expressed mixed perceptions regarding pharmacists’ professional image following the introduction of drive through services, with a substantial proportion expressing concerns about commercial focus, while others perceived a balanced or patient-centered role. The detailed frequencies of the respondents about the image of the community pharmacists were given in Table 2.

**Table 2:** Respondents views on the image of the pharmacists by the introduction of drive through services.

Variables	Strongly Disagree n (%)	Disagree n (%)	Neutral n (%)	Agree n (%)	Strongly agree n (%)
Community pharmacists will appear more concerned with making money than with the health of their patients	9 (2.7)	7 (2.1)	114 (34.0)	151 (45.1)	54 (16.1)
Community pharmacists will have a good balance between the health of patients and the business side of their work	9 (2.7)	13 (3.9)	112 (33.4)	141 (42.1)	60 (17.9)
Community pharmacists will appear more concerned with the health of patients than with the business side of their work	7 (2.1)	26 (7.8)	118 (35.2)	124 (37.0)	60 (17.9)

n = number of respondents.

### 3.3. Public opinions on drive through pharmacy services compared to traditional pharmacies

In this study, 55.2% of the respondents agreed that prescriptions might be filled more quickly at a drive through pharmacy compared to traditional pharmacies, while 13.4% of them disagreed. Regarding the availability of pharmacists at the counter, 56.4% of the respondents agreed that pharmacists might be less available to answer questions using drive through services compared to traditional pharmacies. Additionally, 45.9% of individuals agreed that written information might be less provided at a drive through pharmacy compared to in-store. Furthermore, slightly more than half (51.6%) of the respondents agreed that pharmacists may not be able to explain important points about prescriptions as well while providing drive through services compared to traditional pharmacies.

On the other hand, the majority (87.5%) of respondents agreed that drive through services provide accessibility and convenience to customers more than in-store services, especially during pandemics. 66.5% of them also agreed that unlike in-store services, drive through services are suitable only for refill prescriptions, not for new prescriptions. Table 3 describes the opinions of respondents on drive through pharmacy services compared to traditional pharmacies.

**Table 3:** Opinions of public towards drive through pharmacy services in comparison to normal pharmacies.

Variables	Strongly Disagree n (%)	Disagree n (%)	Neutral n (%)	Agree n (%)	Strongly agree n (%)
The prescription might be filled more quickly in drive through compared to normal pharmacies	6 (1.8)	39 (11.6)	105 (31.3)	134 (40.0)	51 (15.2)
Pharmacists might be less available to answer questions using drive through service compared to normal pharmacies	8 (2.4)	39 (11.6)	99 (29.6)	130 (38.8)	59 (17.6)
Written information might be less supplied using drive through pharmacy service compared to normal pharmacies	11 (3.3)	38 (11.3)	131 (39.1)	103 (30.7)	51 (15.2)
Pharmacists cannot explain important points about prescriptions while providing drive through service compared to that normal pharmacies	14 (4.2)	35 (10.4)	113 (33.7)	125 (37.3)	48 (14.3)
Drive through service provides accessibility and convenience to customers more than the in-store service, especially during any pandemic times	4 (1.2)	9 (2.7)	29 (8.7)	99 (29.6)	194 (57.9)
Unlike in-store service, drive through service is suitable only for refill prescriptions but not for new prescriptions	12 (3.6)	15 (4.5)	85 (25.4)	108 (32.2)	115 (34.3)
Unlike in-store service, drive through service is suitable only for OTC but not for prescriptions medications.	69 (20.6)	82 (24.5)	28 (8.4)	80 (23.9)	76 (22.7)

n = number of respondents, OTC = over the counter.

### 3.4. Perceived advantages towards drive through pharmacy services

**Figure 2.**
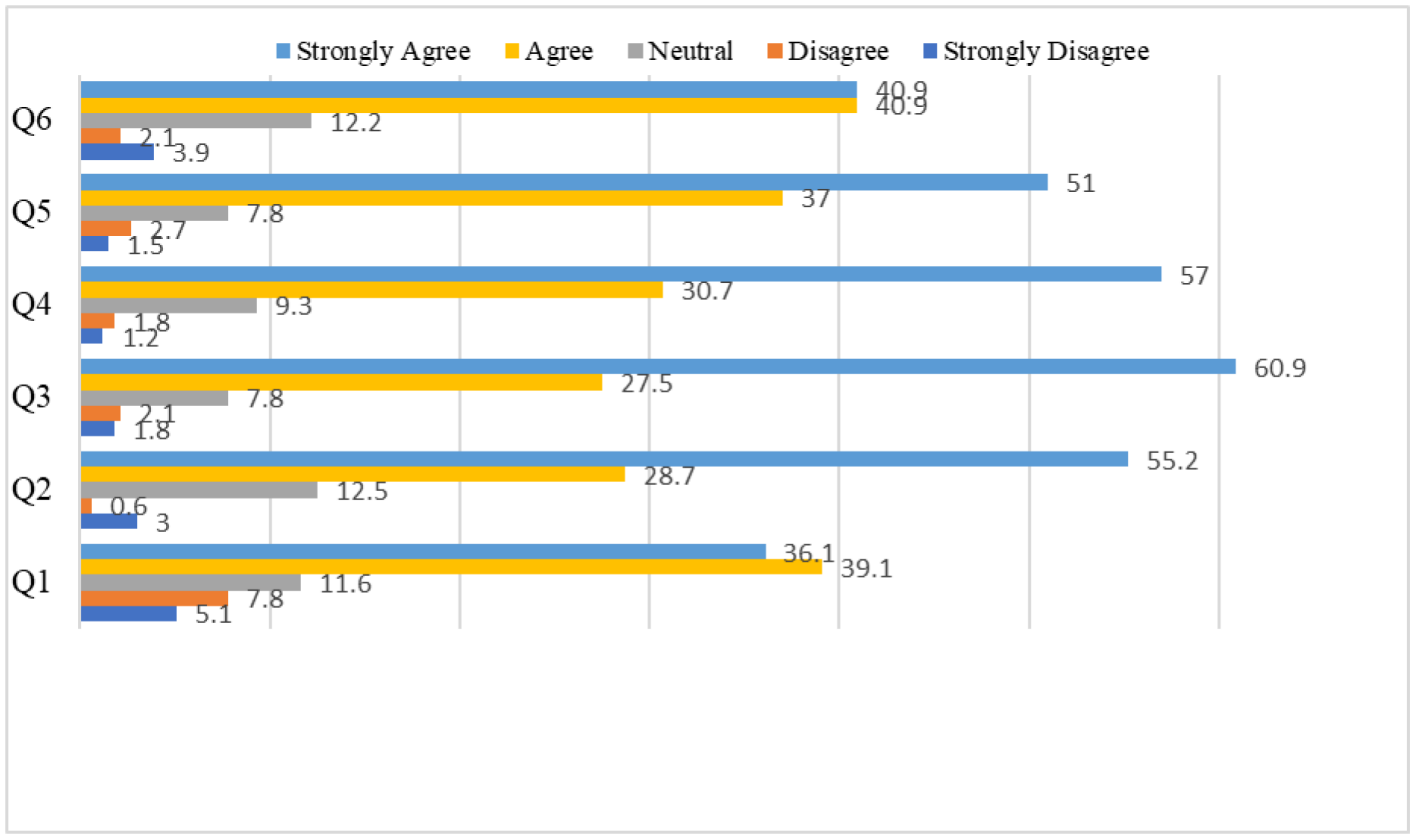
Believed advantages of drive through pharmacy services among respondents.

Most respondents viewed drive through pharmacy services positively, notably in terms of timely drug access, utility during pandemics, enhanced accessibility for the sick, old, and disabled. Many respondents saw drive through services as good for increasing social separation and lowering the chance of disease transmission. Figure 2 and Table S1 summarizes these perceived advantages.

**Figure 3.**
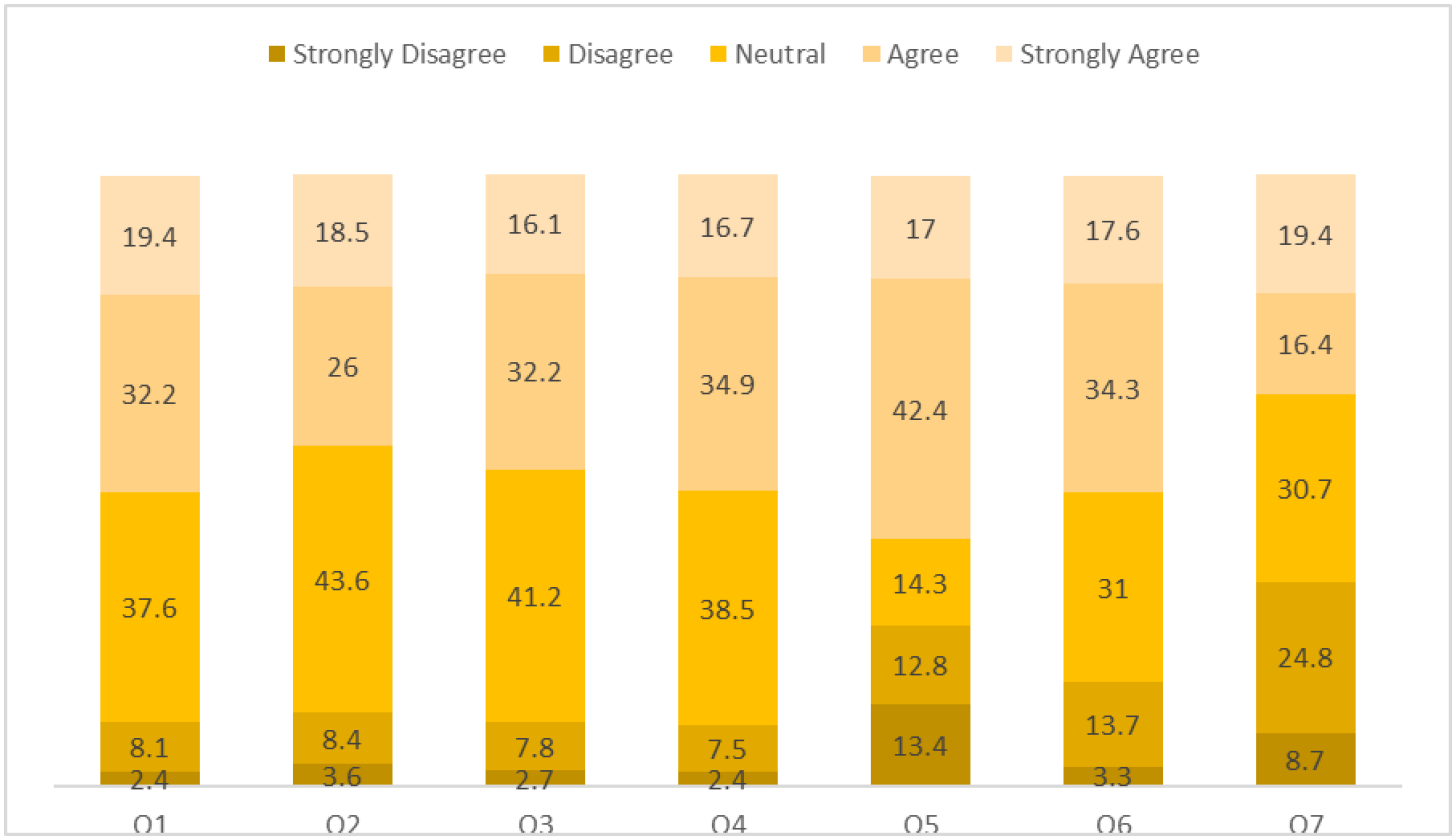
Believed disadvantages of drive through pharmacy services as reported by respondents.

With respect to perceived drawbacks of drive through pharmacy services, respondents expressed concerns related to dispensing accuracy, communication between pharmacists and patients, and the limited provision of medication counseling, particularly written information. Many participants also felt that reliance on drive through services for faster prescription collection could negatively affect the overall quality of pharmacy care and reduce opportunities for meaningful interaction with pharmacists. These perceived disadvantages are illustrated in Figure 3 and Table S2.

**Figure 4.**
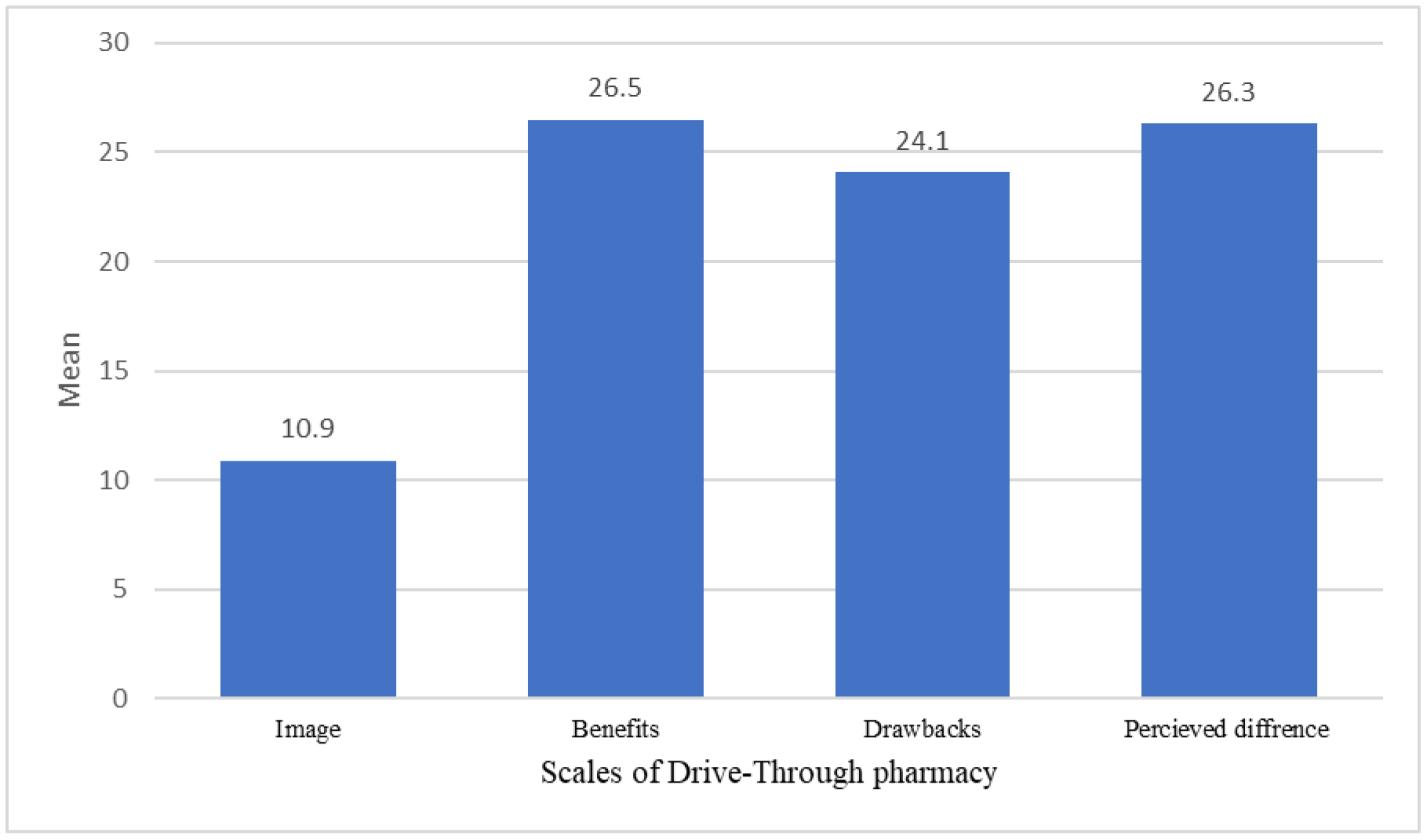
Mean scores of pharmacist image, perceived benefits, and perceived drawbacks of drive through pharmacy services

The mean image of pharmacist was 10.9 ± 2.1 (median = 11), while the mean score for perceived benefits of drive-thru pharmacy were 26.5 ± 4.06 (median = 28), the mean score for perceived drawback was 24.1 ± 5.4 (median = 24), as shown in Figure 4.

Table 4 outlines how perceptions of pharmacist image among respondents vary with different sociodemographic backgrounds. The results show statistically significant variations across gender, age group, marital status, educational level, and employment type (*P* < .05). For example, men expressed slightly more favorable perceptions of pharmacist’s image (mean = 11.18, SD = 2.24) than women (mean = 10.55, SD = 1.82), and this difference was statistically significant (*P* = .004). Age also played a notable role (*P* = .001) respondents aged 18 to 25 demonstrated the most positive perceptions (mean = 11.84, SD = 2.55), whereas individuals older than 46 years held the least favorable perceptions (mean = 9.33, SD = 0.70). In relation to marital status, single respondents reported higher appreciation of pharmacist’s image (mean = 11.55, SD = 2.56) compared to those who were married, divorced, or widowed (*P* = .001). Educational attainment was another significant factor (*P* = .047), with respondents who had completed secondary school yielding the highest perception scores of pharmacist image (mean = 11.29, SD = 1.40). Employment status further influenced perceptions (*P* = .001), homemaker has recorded the most positive ratings (mean = 11.91, SD = 2.63), followed by students (mean = 12.50, SD = 2.12), while employed individuals scored the lowest perceptions score for pharmacist image (mean = 10.50, SD = 1.75) (Table 4).

**Table 4:** Comparison of public perceptions of pharmacist image according to participant’s sociodemographic characteristics.

Characteristics		N	Mean	Mean rank	Median	SD	*P* value
Gender	Male	232	11.18	177.89	11	2.24	.004*
	Female	103	10.55	145.72	10	1.82	
Age	18–25	114	11.84	213.36	12	2.55	.001†
	26–30	64	10.39	138.84	10	1.34	
	31–35	79	10.65	153.61	11	1.81	
	36–40	47	10.40	131.52	10	1.98	
	41–45	22	11.50	187.52	11.5	1.99	
	>46	9	9.33	69.89	9	0.70	
Marital status	Divorced	9	10.33	131.94	10	1.65	.001†
	Married	180	10.63	150.12	10	1.72	
	Single	138	11.55	196.59	11	2.56	
	Widowed	8	10.12	117.69	10	0.99	
Education	Doctorate	51	10.56	141.63	10	1.98	.047†
	Secondary school	44	11.29	188.95	12	1.40	
	University	240	11.02	169.76	11	2.27	
Employment	Employed	175	10.50	146.14	10	1.75	.001†
	Unemployed	37	10.62	139.38	10	1.63	
	Retired	20	11.15	171.93	11	1.89	
	House wife	101	11.91	214.16	12	2.63	
	students	2	12.50	240.25	12.50	2.12	

N = number of respondents, SD = standard deviation.

* Mann–Whitney *U* test.

† Kruskal–Wallis test.

Men tended to express slightly more favorable perceptions about drive through pharmacy benefits (mean = 26.72, SD = 4.29) than women (mean = 26.17, SD = 3.48), and this gender difference reached statistical significance (*P* = .024). Age did not have a notable impact (*P* = .110), though respondents aged 41 to 45 displayed the highest mean score (mean = 28.04, SD = 2.33), indicating a somewhat more positive attitude than other age brackets. Perceptions also varied modestly by marital status (*P* = .290); single respondents (mean = 26.63, SD = 4.67) and widowed respondents (mean = 27.37, SD = 1.59) had slightly higher scores compared to married or divorced individuals. Education level showed a significant effect (*P* = .001). Those with university degrees (mean = 26.80, SD = 4.21) and doctoral qualifications (mean = 26.64, SD = 3.58) held more positive views than respondents with only secondary school education (mean = 25.11, SD = 3.48). Employment type was also associated with notable differences (*P* = .004). Table 5 summarizes how respondents views regarding the advantages of drive through pharmacy services differ based on their sociodemographic features.

**Table 5:** Differences in perceptions of benefits of drive through pharmacy and statistical comparisons of the participants according to their sociodemographic characteristics.

Characteristics		N	Mean	Mean rank	Median	SD	*P* value
Gender	Male	232	26.72	116.50	28.00	4.29	.024*
	Female	103	26.17	52	27.00	3.48	
Age	18–25	114	26.64	176.57	28.00	4.59	.110†
	26–30	64	26.46	155.63	27.00	3.19	
	31–35	79	25.96	151.33	27.00	4.26	
	36–40	47	26.93	182.50	28.00	3.99	
	41–45	22	28.04	201.27	28.50	2.33	
	>46	9	25.55	136.67	28.00	3.90	
Marital status	Divorced	9	24.55	129.72	24.00	5.27	.290†
	Married	180	26.56	161.86	27.50	3.53	
	Single	138	26.63	178.29	28.00	4.67	
	Widowed	8	27.37	171.63	28.00	1.59	
Education	Doctorate	51	26.64	166.08	28.00	3.58	.001†
	Secondary school	44	25.11	119.83	26.00	3.48	
	University	240	26.80	177.24	28.00	4.21	
Employment	Employed	175	26.09	150.61	27.00	3.80	004†
	Unemployed	37	26.62	171.24	28.00	3.94	
	Retired	20	27.85	201.10	29.00	2.71	
	House wife	101	27.09	191.70	28.00	4.68	
	students	2	25.50	101.75	25.50	0.70	

N = number of respondents, SD = standard deviation.

* Mann–Whitney *U* test.

† Kruskal–Wallis test.

Table 6 explores how perceptions of the disadvantages associated with drive through pharmacy services differ among respondents based on their sociodemographic factors. The analysis of 335 respondents revealed statistically significant variations across gender, age, marital status, and employment categories (*P* < .05), while education level did not show a significant impact. Gender-based differences were notable (*P* = .013), with men reporting higher average scores for perceived drawbacks (mean = 24.75, SD = 5.58) compared with women (mean = 22.69, SD = 4.69). Age-related differences were also significant (*P* = .001). Respondents aged 18 to 25 demonstrated the greatest concern regarding potential drawbacks (mean = 26.77, SD = 6.64), while those aged 26 to 30 showed the lowest mean score (mean = 21.81, SD = 3.85), suggesting younger adults were more critical of the system. In terms of marital status (*P* = .001), single individuals exhibited stronger perceptions of drawbacks (mean = 26.14, SD = 6.43) than married, divorced, or widowed respondents. Educational attainment, however, did not significantly influence perceptions (*P* = .369), as mean scores remained relatively consistent across doctorate (mean = 24.13), university (mean = 24.30), and secondary school graduates (mean = 23.11). Employment type presented a significant difference (*P* = .001) (Table 6)

**Table 6:** Differences in perceptions of drawbacks of drive-through pharmacy and statistical comparisons of the participants according to their sociodemographic characteristics.

Characteristics		N	Mean	Mean rank	Median	SD	*P* value
Gender	Male	232	24.75	176.72	24.00	5.58	**.013** *
	Female	103	22.69	148.36	23.00	4.69	
Age	18-25	114	26.77	215.82	26.00	6.64	**.001** †
	26–30	64	21.81	122.71	22.00	3.85	
	31–35	79	22.70	138.39	22.00	3.69	
	36–40	47	23.00	155.57	24.00	4.02	
	41–45	22	24.59	176.47	23.00	4.21	
	46	9	24.22	188.50	25.00	6.09	
Marital status	Divorced	9	23.44	180.78	24.00	5.00	**.001** †
	Married	180	22.61	138.44	23.00	3.96	
	Single	138	26.14	204.91	26.00	6.43	
	Widowed	8	24.00	181.88	25.50	3.77	
Education	Doctorate	51	24.13	178.98	24.00	5.06	**.369** †
	Secondary school	44	23.11	151.28	23.00	3.27	
	University	240	24.30	168.73	24.00	5.77	
Employment	Employed	175	22.22	131.72	22.00	4.11	**.001** †
	Unemployed	37	23.05	155.08	24.00	4.10	
	Retired	20	24.90	196.58	25.00	2.48	
	Pharmacy students	101	27.54	227.96	27.00	6.41	
	Ophthalmic students	2	30.00	268.00	30.00	5.65	

N = number of respondents, SD = standard deviation.

* Mann–Whitney *U* test.

† Kruskal–Wallis test.

## 4. Discussion

The expanding role of pharmacists from traditional dispensing toward broader patient-centered care is now widely recognized, attracting greater attention from both the public and other healthcare professionals. Despite their established position as healthcare providers, pharmacists are still often perceived through multiple lenses: as medicine vendors, drug experts, tradespeople, and business operators. This multifaceted perception reflects the unique dual nature of community pharmacy practice, where clinical responsibilities coexist with commercial functions.^[18]^ Although pharmacists are widely regarded as medication experts, many respondents did not fully perceive them in this role.^[18]^ Previous findings have also shown that the public’s understanding of the care provided by pharmacists and their broader professional responsibilities remains limited. This suggests that despite their clinical training and expanding scope of practice, pharmacists are not always recognized by consumers as comprehensive healthcare providers in today’s healthcare system.^[22]^

Although the current findings revealed mixed perceptions of the pharmacist image among the public after the introduction of drive through pharmacy services, there were some interesting trends. For example, a considerable proportion of individuals perceived that pharmacists appear more concerned with making money than with the health of their patients, while a substantial number disagreed with this perception. However, respondents also agreed that pharmacists have struck a good balance between patient health and the business side of their work with the introduction of drive through pharmacy services. Additionally, a notable segment of respondents agreed that pharmacists appear more concerned with the health of patients than with the business side of their work. These findings were comparable to earlier research^[17]^ where author revealed that 68.3% of the respondents agreed that pharmacist have a good balance between managing patient health and business side of their work.^[17]^ In addition, 56.3% of the respondents in previous study agreed that pharmacists were more concerned with the health of patients rather than their pharmacy business.^[17]^

Despite these results, earlier research indicates that 81% of adults in Saudi Arabia felt that pharmacists respected them during dispensing, gave those clear instructions on how to take their medications, and most of them trusted the advice of pharmacists regarding prescription drugs^[9]^. This suggests that pharmacists have a good image among the Saudi public. Additionally, individuals in Saudi Arabia expressed tremendous satisfaction with the expertise and pharmaceutical services provided by pharmacists^[9]^. Similarly, another study conducted in Saudi Arabia found that the availability of pharmacists in the pharmacy, their competence, their promptness in providing service, and the amount of time they spend counseling customers all had an impact on the public’s perception of the pharmacist’s image^[9]^. Although literature in well-developed countries has shown that consumers are unaware that pharmacists are trained to provide additional services such as medication inquiry services and drug therapy optimization, the public expects personalized services from pharmacists.^[22]^ These findings suggest that individuals in Saudi Arabia have a positive perception and image of pharmacists. However, in some aspects, individuals lack knowledge and understanding of the services offered by pharmacists. Therefore, creating awareness of the pharmacist’s role among the public is necessary.

Regarding the beneficial effects of drive through pharmacy services, findings revealed that 83.9% of respondents agreed that drive through pharmacy services were useful during different pandemics. Additionally, 75.2% indicated that getting prescriptions on time and immediately was important, and 88.4% stated that the service’s benefit was that it might help sick patients, the elderly, or those with disabilities. Similar findings have been observed in other countries.^[10,12,13]^ For example, Abu Farha et al study of Jordanians found that the most frequently mentioned advantages of drive through pharmacy services were their ability to serve the ill, the elderly, disabled patients, and mothers traveling with children.^[12]^

Similarly, another recent study conducted among Malaysians by Ababneh et al found that drive through pharmacy services might also be useful during various pandemics and quarantine periods to improve social distance and lessen the spread of various illnesses and viruses.^[10]^ Likewise, a study conducted in 2023 by Lamin et al found that 94.5% of respondents thought that drive through services were beneficial for patients who are busy with their daily routine and reduce patients’ waiting times to pick up their medications. Additionally, 87.2% of respondents thought that drive through services were beneficial for senior citizens or caregivers.^[23]^

In this study younger and single respondent, reported greater concerns about drive through services. They also indicated more positive perceptions of pharmacists’ image. Given their less frequent interaction with other aspects of the healthcare system, younger and single people frequently rely on pharmacies as easily accessible sites of healthcare.^[24]^ They may have a better understanding of the functions of pharmacists because of their increased exposure to contemporary public health messaging and preference for convenience.^[24]^ However, the same group might appreciate comprehensive counseling and clear communication more, which they would feel are lost in a fast-paced drive through setting. Such nuanced expectations may help to explain why cautious attitudes about potential downsides of drive through coexist with positive opinions of pharmacists’ overall, image. Sociodemographic influences were also evident among other groups. For instance, homemakers and students had more positive perceptions of pharmacists’ image compared to employed individuals. This difference may be due to variations in time availability, as those not constrained by rigid work hours have more opportunities for meaningful interactions with pharmacists. Conversely, employed individuals may perceive pharmacy visits as hurried transactions focused on efficiency, potentially diminishing their perceptions of professional engagement.

Although drive through pharmacy services have many advantages,^[20,25]^ respondents to the current study also identified drawbacks. Of these, 51.9% claimed that drive through pharmacy services could lead to dispensing errors, while 44.5% said that patient-pharmacist communication errors could be a contributing factor to dispensing errors. Furthermore, 51.6% of respondents indicated that drive through pharmacy services are inconvenient for providing patients with drug information and counseling. When Ababneh et al surveyed Malaysians, they discovered comparable outcomes.^[19]^ Of them, 63% believed that drive through pharmacies, offering quick service, could result in dispensing errors. 68.5% cited communication errors as another disadvantage, 57.8% mentioned the drawback of additional funding for drive through windows, and 61.6% said that providing patients with drug information and counseling is inconvenient and a major drawback of drive through pharmacy.^[19]^ However, a survey of pharmacists found that drive through services can negatively impact the reputation of the pharmacy industry and cause pharmacists to feel more like fast food employees than pharmacists.^[21]^ Thus, it is necessary to comprehend the main issues surrounding the delivery of extended and drive through pharmacy services and to enhance pharmacists’ abilities through additional training programs in order to effectively provide such services.

This study has several limitations that should be considered when interpreting the findings. First, the use of online questionnaires and convenience sampling methods may introduce selection bias. Individuals who are more active on social media or interested in healthcare topics may have been more likely to participate. Second, the study relied on self-reported data, which are subject to recall and social desirability biases, potentially influencing respondents’ answers. Additionally, the generalizability of the findings is limited as the sample was drawn from a single geographic region in Saudi Arabia and included a higher proportion of male participants with a relatively higher mean age. These demographic characteristics may have influenced perceptions of pharmacists and drive through pharmacy services, as attitudes toward healthcare delivery models can vary by gender and age. Therefore, the results may not fully represent the views of younger individuals, females, or populations in other regions. Another potential limitation of this study is that the results are unadjusted, as no multivariable analyses were performed, and possible interactions with demographic factors such as age, education, and employment were not accounted for. Future studies using more diverse and representative samples and probability-based sampling methods are recommended to validate and extend these findings.

## 5. Conclusion

The findings indicate that pharmacists are generally regarded as committed healthcare professionals who attempt to balance clinical responsibilities with the commercial realities of practice. drive through services were viewed as particularly valuable in enhancing convenience and access to medications, especially for individuals with mobility limitations and during periods when minimizing direct contact is important. At the same time, participants expressed concerns about whether this model may compromise the depth of communication, the quality of counseling, and the safety of medication dispensing. These mixed perceptions underline the need to strengthen communication strategies and ensure that service efficiency does not come at the expense of patient-centered care. Structured training initiatives, workflow adjustments, and clear operational standards may help mitigate perceived shortcomings and reinforce public confidence in pharmacists’ expanding roles. Nevertheless, the conclusions drawn from this study should be interpreted carefully. The use of convenience sampling and self-administered online questionnaires may limit the representativeness of the sample and introduce response bias. In addition, the analysis did not control for potential confounding variables, which restricts interpretation of observed differences between demographic groups. Therefore, the findings reflect the perspectives of the surveyed participants rather than the broader population of Riyadh. Further research employing more rigorous sampling strategies and analytical methods is warranted to provide stronger evidence and to guide the development of standardized policies for drive through pharmacy services.

## Acknowledgments

The authors of this study extend their appreciation to the Ongoing Research Funding program (ORF-2026-1099), King Saud University, Riyadh 11451, Saudi Arabia, for supporting this study and for funding this work.

## Author contributions

**Conceptualization:** Raeed Alanazi, Muteb Abdullah Saqer Alfutaymani, Naji Alqahtani, Wajid Syed.

**Data curation:** Raeed Alanazi, Mohammed Almutairi, Muteb Abdullah Saqer Alfutaymani, Marzouq Daije S. Alotaibi, Wajid Syed.

**Formal analysis:** Raeed Alanazi, Mohammed Almutairi, Mohammad K. Alharbi, Muteb Abdullah Saqer Alfutaymani, Marzouq Daije S. Alotaibi, Naji Alqahtani, Wajid Syed.

**Funding acquisition:** Raeed Alanazi, Mohammed Almutairi, Mohammad K. Alharbi, Naji Alqahtani, Wajid Syed.

**Investigation:** Raeed Alanazi, Mohammed Almutairi, Marzouq Daije S. Alotaibi, Naji Alqahtani.

**Methodology:** Raeed Alanazi, Mohammad K. Alharbi, Muteb Abdullah Saqer Alfutaymani, Naji Alqahtani.

**Project administration:** Raeed Alanazi, Mohammed Almutairi, Marzouq Daije S. Alotaibi, Naji Alqahtani.

**Resources:** Mohammed Almutairi, Mohammad K. Alharbi, Muteb Abdullah Saqer Alfutaymani, Marzouq Daije S. Alotaibi, Naji Alqahtani, Wajid Syed.

**Software:** Mohammed Almutairi, Mohammad K. Alharbi, Muteb Abdullah Saqer Alfutaymani, Marzouq Daije S. Alotaibi, Naji Alqahtani.

**Supervision:** Mohammed Almutairi, Mohammad K. Alharbi, Naji Alqahtani.

**Validation:** Mohammed Almutairi, Mohammad K. Alharbi, Marzouq Daije S. Alotaibi, Naji Alqahtani.

**Visualization:** Raeed Alanazi, Mohammed Almutairi, Mohammad K. Alharbi, Wajid Syed.

**Writing—original draft:** Raeed Alanazi, Wajid Syed.

**Writing—review & editing:** Raeed Alanazi, Mohammed Almutairi, Mohammad K. Alharbi, Muteb Abdullah Saqer Alfutaymani, Marzouq Daije S. Alotaibi, Wajid Syed.





## References

[1] Atkinson J. Advances in pharmacy practice: a look towards the future. Pharmacy (Basel). 2022;10:125. 36287446 10.3390/pharmacy10050125 PMC9608826

[2] Diri RM. The impact of COVID-19 outbreak on reassessing the need for drive thru community pharmacy: cross-sectional study. J Microsc Ultrastruct. 2020;8:162–4. 33623741 10.4103/JMAU.JMAU_65_20 PMC7883499

[3] Ali Thorakkattil S Madathil H Abideen Parakkal S. Advancements in ambulatory care pharmacy practice in Saudi Arabia: a comprehensive review of innovations and best practices at Johns Hopkins Aramco Healthcare. Saudi Pharm J. 2024;32:102170. 39308955 10.1016/j.jsps.2024.102170 PMC11415966

[4] Asiri IM Alrastal DY Alaqeel RK. A survey of drive-thru pharmacy services: Evaluating the acceptance and perspectives of community pharmacists in Saudi Arabia. Saudi Pharm J. 2024;32:101924. 38226348 10.1016/j.jsps.2023.101924 PMC10788628

[5] Myers A. Drive-through businesses. Am Bus Hist Civ Libert. 2011.

[6] Padilla JA Faller EM. Drive-thru community pharmacy in the “new normal era”: an innovation in pharmaceutical services and its socio-economic impact. GSC Biol Pharm Sci. 2022;18:137–54.

[7] Radwan R. Hospital drive-through in Saudi Arabia is sweet medicine for patients. Arab News. 2020. https://arab.news/j9akz. Accessed May 18, 2026.

[8] Syed W Basil AA-RM. Assessment of awareness, perceptions, and opinions toward artificial intelligence among healthcare students in Riyadh, Saudi Arabia. Medicina (Kaunas). 2023;59:828. 37241062 10.3390/medicina59050828 PMC10221309

[9] Wajid S. A survey on pharmacist opinion about pharmaceutical care in Saudi Arabia. Asian J Pharm. 2015;9:4.

[10] Tadesse YB Sendekie AK Mekonnen BA Denberu FG Kassaw AT. Pharmacists’ medication counseling practices and knowledge and satisfaction of patients with an outpatient hospital pharmacy service. Inquiry. 2023;60:469580231219457. 38131171 10.1177/00469580231219457 PMC10748552

[11] Naqvi AA Umair Khan M Nguyen H Karim L Said A Nnadi A. Exploring pharmacists’ perceptions of their current role in mental health trusts in england: a qualitative study. Healthcare. 2025;13:2602. 41154280 10.3390/healthcare13202602 PMC12563904

[12] Ciliberti R Bonsignore A. New integrations in patient care: the role of the pharmacist between couselling and medication adherence. J Prev Med Hyg. 2025;66:E38–44. 40756186 10.15167/2421-4248/jpmh2025.66.1.3222 PMC12312715

[13] Esmalipour R Salary P Shojaei A. Trust-building in the pharmacist-patient relationship: a qualitative study. Iran J Pharm Res. 2021;20:20–30. 34903966 10.22037/ijpr.2020.114113.14675 PMC8653675

[14] Rasheed MK Alqasoumi A Hasan SS Babar Z-U-D. The community pharmacy practice change towards patient-centered care in Saudi Arabia: a qualitative perspective. J Pharm Policy Pract. 2020;13:59. 32944258 10.1186/s40545-020-00267-7 PMC7488651

[15] Thorakkattil SA Parakkal SA Mohammed Salim KT. Improving patient safety and access to healthcare: the role of pharmacist-managed clinics in optimizing therapeutic outcomes. Explor Res Clin Soc Pharm. 2024;16:100527. 39469652 10.1016/j.rcsop.2024.100527 PMC11513600

[16] Black G. Patient encounters… pharmacists have rights too! S Afr Pharm J. 2022;89:47–8.

[17] Ababneh BF Ong SC Mahmoud F Alsaloumi L Hussain R. Attitudes, awareness, and perceptions of general public and pharmacists toward the extended community pharmacy services and drive-thru pharmacy services: a systematic review. J Pharm Policy Pract. 2023;16:37. 36864499 10.1186/s40545-023-00525-4 PMC9979876

[18] El-Kholy AA Abdelaal K Alqhtani H Abdel-Wahab BA Abdel-Latif MM. Public perceptions of community pharmacists and satisfaction with pharmacy services in Al-Madinah City, Saudi Arabia: a cross-sectional study. Medicina. 2022;58:432. 35334609 10.3390/medicina58030432 PMC8954639

[19] Ababneh BF Ong SC Hussain R. Awareness, attitudes, and perceptions of drive-thru community pharmacy services among the general public in Malaysia during COVID-19: A cross-sectional study. PLoS One. 2023;18:e0282991. 36897873 10.1371/journal.pone.0282991 PMC10004521

[20] Bakarman SS Syed W Alharbi MK Bashatah A Al-Rawi MBA. Public perceptions and attitudes of drive-through pharmacy services: Insights from a cross-sectional survey in Saudi Arabia. Medicine (Baltimore). 2025;104:e41118. 39792718 10.1097/MD.0000000000041118 PMC11730408

[21] Farha RA Hammour KA Alefishat E Alsaeed H Alma’aiah S. Drive-thru pharmacy service: assessments of awareness, perception, and barriers among pharmacists in Jordan. Saudi Pharm J. 2017;25:1231–6. 29204073 10.1016/j.jsps.2017.09.008 PMC5688229

[22] Low HMM Lai YF. Understanding and expectations toward pharmaceutical care among patients, caregivers, and pharmacy service providers: a qualitative study. Eur J Hosp Pharm. 2020;27:25–30. 32064085 10.1136/ejhpharm-2017-001415 PMC6992973

[23] Lamin RAC Jamil AFM Anuar FAF. Pharmacy staff perceptions and opinions on the drive-through pharmacy service during COVID-19 pandemic at a university teaching hospital in Malaysia. Malays J Pharm. 2023;9:11–5.

[24] Valliant SN Burbage SC Pathak S Urick BY. Pharmacists as accessible health care providers: quantifying the opportunity. J Manag Care Spec Pharm. 2022;28:85–90. 34949110 10.18553/jmcp.2022.28.1.85 PMC8890748

[25] Hussain R Dawoud DM Babar ZU. Drive-thru pharmacy services: a way forward to combat COVID-19 pandemic. Res Social Adm Pharm. 2021;17:1920–4. 32792322 10.1016/j.sapharm.2020.07.015 PMC7373674

