# Supplementary material for: Assessing the image of pharmacists and perceived public prospective on drive through pharmacy services in Saudi Arabia: A cross-sectional study
Source: Medicine (Baltimore). 2026 May 29;105(22):e48982. doi: 10.1097/MD.0000000000048982 (PMC13225483; doi:10.1097/MD.0000000000048982)
Supplement: Supplementary file 1 [file medi-105-e48982-s001.doc]

**Supplementary Table-2** Believed drawbacks of drive-thru pharmacy services

|  | **Variables** |
| --- | --- |
| Q1 | Drive-thru pharmacy service may contribute to dispensing errors due to the fast service provided. |
| Q2 | Drive-thru pharmacy service may contribute to communication errors between the patient and pharmacist |
| Q3 | Drive-thru pharmacy service may need extra money to offer drive-thru windows. |
| Q4 | Drive-thru pharmacy service is not convenient in providing drug information/counselling to patients (especially written information). |
| Q5 | Getting prescriptions dispensed as quickly as possible using drive-thru community pharmacy service, the quality of pharmacy service will drop |
| Q6 | Drive-thru pharmacy service restricts the opportunity for interaction with the pharmacist because the customer feels they can’t ask questions while they’re being hurried through. |
| Q7 | Drive-thru pharmacy service restricts the opportunity for interaction with the pharmacist because the pharmacist will not be able to offer any level of interaction. |
