# Supplementary material for: Assessing the image of pharmacists and perceived public prospective on drive through pharmacy services in Saudi Arabia: A cross-sectional study
Source: Medicine (Baltimore). 2026 May 29;105(22):e48982. doi: 10.1097/MD.0000000000048982 (PMC13225483; doi:10.1097/MD.0000000000048982)
Supplement: Supplementary file 2 [file medi-105-e48982-s002.docx]

**Supplementary Table 1 -Believed benefits of drive-thru pharmacy services**

|  | **Variables** |
| --- | --- |
| Q1 | Drive-thru pharmacy service may help me get my medications on time without delay |
| Q2 | Drive-thru pharmacy helpful during various pandemics |
| Q3 | Drive-thru pharmacy service has the advantage of serving sick patients, elderly, or disabled people |
| Q4 | Drive-thru pharmacy service enhances social distancing and reduces the chances of spreading infections. |
| Q5 | Drive-thru pharmacy service reduces the pressure on health care centers |
| Q6 | Drive-thru pharmacy service is needed to be implemented in most community pharmacies during COVID-19 time or even later on for getting medications or supplies. |
